# Calculation and Evaluation of Carbon Footprint in Mulberry Production: A Case of Haining in China

**DOI:** 10.3390/ijerph17041339

**Published:** 2020-02-19

**Authors:** Yi Li, Yi Wang, Qing He, Yongliang Yang

**Affiliations:** 1 East China Sea Institute/Center for Ecological Civilization of Yangtze River Delta, Ningbo University, Ningbo 315211, China; liyi1@nbu.edu.cn; 2 Fashion Department of International United Faculty between Ningbo University and University of Angers/Faculty of Tourism and Culture, Ningbo University, Ningbo 315201, China; 3 School of Economics and Management, Zhejiang Sci-Tech University, Hangzhou 310018, China; 2016333503068@mails.zstu.edu.cn; 4 Fashion Institute/Silk and Fashion Culture Research Center of Zhejiang Province, Zhejiang Sci-Tech University, Hangzhou 310018, China; 2018328420036@mails.zstu.edu.cn; 5 Ecological Civilization Research Center of Zhejiang Province, Zhejiang Sci-Tech University, Hangzhou 310018, China

**Keywords:** mulberry planting, carbon footprint, accounting, life cycle assessment

## Abstract

Carbon footprint refers to the greenhouse gas emissions of an activity during the whole life cycle or a specific period of time. Mulberry is an important cash crop. Thus, establishing a standardized accounting method for the carbon footprint of mulberry production and analyzing its carbon emission scenarios is important in correctly understanding the impact of mulberry production on the environment. Using the life cycle assessment method and on the basis of the statistical data of mulberry production of urban farmers in Haining City, China, in 2014–2016, this study calculates and evaluates the carbon footprint of mulberry production. Results show the following. (1) Indirect carbon emissions is the main part of total carbon emissions, accounting for 85%–88% of total carbon emission, and industrial inputs (fertilizers and pesticides) are the main cause of carbon emissions. (2) The total carbon emissions per hectare in 2016 (6550.73 kgce/hm^2^) rose relative to the 2015 data (5617.92 kgce/hm^2^ at least in 2014) (5729.64 kgce/hm^2^). The output value of mulberry in spring was greater than that in summer and autumn, and the production efficiency of mulberry carbon in spring was higher than that in summer and autumn. The ecological environment of the mulberry production industry can be improved by increasing the resources of carbon sequestration and reducing the source of production input. (3) In general, the photosynthetic carbon sink of mulberry is greater than the total carbon emission and presents a positive externality to the ecological environment.

## 1. Introduction

China is now facing a veritable environmental challenge [1]. Climate change is one of the most serious environmental problems that human beings are facing today. The excessive emission of greenhouse gases is the main cause of climate change. Carbon dioxide, in particular, is the most dominant greenhouse gas, accounting for 60% of the total contribution to the greenhouse effect [2]. China clearly matters when it comes to global efforts to mitigate climate change and any successful international efforts to stabilize greenhouse gases (GHG) emissions must inevitably include the country [3]. Since the 21st century, China has been facing huge pressure to reduce its greenhouse gas emissions [4,5]. To change this situation and transform into a large green economy, in 2009, China proposed to reduce its carbon dioxide emissions per unit GDP by 40%–45% by 2020 relative to the 2005 data. In 2015, China further proposed to reach the peak carbon dioxide emissions by 2030 and strive to reach such goal as soon as possible. The peak and carbon intensity in 2015 are 60%–65% lower than those in 2005.

China attaches great importance to tackling climate change and actively and effectively controlling greenhouse gas emissions. According to the China Greenhouse Gas Bulletin 2018, the annual average CO_2_ concentrations of the world and China in 2017 were 402.2 ± 2.8 and 405.0 ± 3.0 ppm, respectively, representing an increase of 2.2 and 2.6 ppm from 2016, respectively. These values were basically the same as the annual average absolute increases (2.2 and 2.4 ppm) of the world and China in the past eight years (2010–2017). On August 30, 2019, the Ministry of Ecology and Environment of China held a press conference and pointed out that China’s carbon emission intensity in 2018 was 45.8% lower than that in 2005. This report indicates the rapid growth of greenhouse gas emissions and serves as a solid foundation for the realisation of the 13th Five-Year Plan to tackle climate change and the implementation of the national independent contribution by 2030. China’s carbon emission intensity has been effectively alleviated.

Agriculture is not only a source of greenhouse gas emissions but also the industry which is most vulnerable to the impact of climate change [6]. A strong coupling relationship is observed between agricultural production and greenhouse gas emissions [7]. China’s agricultural carbon emissions account for approximately 17% of the total global carbon emissions [8]. At the same time, China’s agricultural carbon emissions show an obvious spatial imbalance [9] and ladder differences. The major agricultural provinces in the Middle Eastern region are China’s high emission areas, followed by the central and western regions; the non-agricultural cities or the western backward regions are China’s low emission areas [10]. At the same time, through the measurement and analysis of agricultural carbon emissions of China’s main grain producing areas, it is found that the total agricultural carbon emissions of each region in the main grain producing areas tend to increase, but the carbon emission intensity is in a downward trend [11].

Low carbon agriculture is one of the objectives of modern agricultural development. Therefore, the accounting of carbon emissions from agriculture and the analysis of its carbon emission scenarios are the basis for the implementation of low carbon agricultural technology transformation, carbon trading, carbon labelling, and other management measures. Carbon footprint accounting and evaluation have become one of the hot spots in low carbon agriculture research [12,13,14,15]. Carbon footprint comes from ecological footprint. Wackernagel and Rees used ecological footprint to describe the ecological impact caused by human production or consumption [16]. Carbon footprint refers to the greenhouse gas emissions generated by an activity in the whole life cycle or in a specific time [17,18,19,20,21]. The agricultural carbon footprint systematically evaluates the total direct and indirect carbon emissions caused by human factors in the process of agricultural cultivation, fertilization, and harvest. It also quantitatively measures the impact of agricultural production activities on the greenhouse effect.

The calculation and evaluation of carbon footprint are carried out using life cycle assessment (LCA). LCA is a bottom–up accounting method, which can be applied to the relative microscale accounting and evaluation of carbon footprint. It can cover all stages of production and the entire process, from production source to consumption and the disposal of production waste. LCA is a ‘cradle to grave’ accounting method [22,23]. The most authoritative definition of LCA is that of the International Standard Organization (ISO) and International Society for Environmental Toxicology and Chemistry (SETAC). The ISO [24] defines LCA as a method of accounting and evaluating the potential impact of all inputs and outputs on the environment of a product (or) service system in its whole life cycle. The SETAC [25] defines LCA as a method for identifying and quantifying the pressure of products, production processes and activities on the environment.

Scholars at home and abroad use LCA to calculate and analyze the carbon footprint of agricultural production [26,27,28,29,30,31,32,33,34,35] and suggest countermeasures and suggestions on how to realize the low carbon development of agriculture. A number of them have performed in-depth research on the main sources of agricultural carbon emissions. For example, Huang Zuhui et al. [36] took Zhejiang Province as an example, applied the layered input–output LCA method to calculate the net carbon footprint of regional agriculture and found that the proportion of greenhouse gases emitted by planting and breeding activities is small; direct and indirect carbon emissions from agricultural energy, carbon emissions from the whole life cycle of industrial inputs and the final disposal of agricultural wastes were identified as three of the most important sources of agricultural greenhouse gases. Zhang Guangsheng et al. [37] constructed China’s agricultural carbon emission measurement system by using the LCA method and revealed that agricultural carbon emissions gradually develop from planting and breeding natural sources to a situation where the proportions of energy and chemicals and natural sources are roughly the same. Considering LCA, some scholars have compared and analyzed the carbon footprint of agricultural products. For example, Xu Shuya et al. [38] found that the carbon footprint of oats during planting is considerably lower than that of corn, rice, and wheat. Zhu Qiang et al. [39] found that the carbon emission per kilogram of organic rice is significantly higher than that of non-organic rice by comparing the carbon footprint of organic rice with that of other ordinary rice. Many scholars have also studied the carbon footprint of agricultural production systems. Marie et al. [40] compared the carbon footprints of organic and traditional rotation plant systems by LCA and pointed out that in terms of reducing greenhouse gas emissions, a rotation system should be selected to grow legume crops. Song Bo et al. [33] found that the photosynthetic carbon sink of a greenhouse vegetable production system is greater than the total carbon emission, which has a positive externality to the ecological environment. Duan Huaping [41] estimated the carbon emission, carbon absorption and carbon footprint of China’s farmland ecosystem and observed an upward trend.

Mulberry is not only an important economic crop, but also an important material basis for silk production and the manufacture of textiles and clothing. In the production process of mulberry trees, greenhouse gas emissions are produced to varying degrees. However, no scholars have yet accounted for the carbon footprint of mulberry production. Haining City, located in Taihu Lake Basin, is an important base for mulberry production and silk production in China. Therefore, this paper takes Haining City of Zhejiang Province as an example to study the carbon footprint of mulberry production stage. In this paper, the carbon emission of mulberry and its related indicators are calculated, the carbon emission scenarios are analyzed, the impact of mulberry production on the environment is deeply understood, and it is of great significance to seek the green production way of sericulture agriculture. In this paper, LCA method is used to calculate and analyze the carbon footprint of mulberry production stage, and a set of standardized carbon footprint accounting system for mulberry production is constructed. The carbon footprint of mulberry production stage is comprehensively evaluated from the perspective of ecological function, social function and economic function. The results should provide a reference for accounting and evaluating the carbon footprint of mulberry production stage and lay a foundation for the final realization of low carbon planting.

This paper is organized as follows. Section 2 offers an overview of the main data and methodology this paper uses to calculate and evaluate the carbon footprint of mulberry trees; and Section 3 analyzes the carbon footprint of mulberry production in 2014; then it compares the carbon footprint of mulberry production in different years. Section 4 concludes the study.

## 2. Data and Methodology

### 2.1. Data

The data used in this research are from the statistical survey of mulberry planting farmers in the jurisdiction (including villages and towns such as Zhouwangmiao, Yunlong, Hutang, Minfeng, and Boru) of Haining Agricultural Economic Bureau of China. The planting mode in this area is based on farmers’ families, they are under the guidance of technicians engaged in standardized planting, fertilization, irrigation, and branch digging. The survey data involves 15 mulberry growers in one year, with a total planting area of 1.764 hm^2^. The minimum planting area of mulberry is 0.0667 hm^2^, and the maximum is 0.2333 hm^2^; the average density is 12,150 plants/hm^2^. Considering the difference in technical levels, we sum up and average the data on labor, chemical fertilizers, pesticides, and manure according to the investigation. The data collection period is from January 2014 to December 2016.

### 2.2. Carbon Footprint Accounting Method

Under the LCA method, the carbon footprint of the mulberry production stage is calculated as follows: set the system boundary, define the greenhouse gases, establish the calculation formula and interpret the result.

#### 2.2.1. Setting System Boundaries

**Figure 1:**
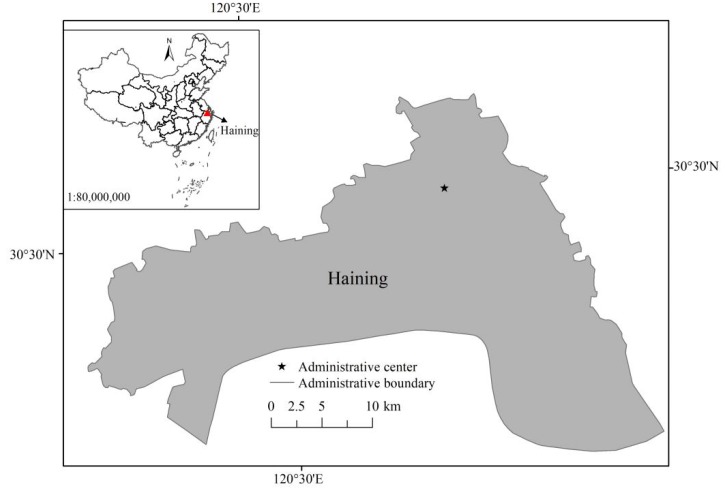
Location of Haining in China.

In this study, the system boundary is set as the whole process of the agricultural production stage of adult mulberry that has been put into production. According to the statistical caliber of the mulberry survey data of the Haining Agricultural Economic Bureau of China, the whole process of mulberry production is divided into two stages: spring (from March 20 to May 20), summer and autumn (from June 10 to October 20). The harvest period of summer and autumn includes two stages: mid-autumn mulberry and late autumn mulberry. The two growth stages of mulberry in a year need the input of chemical fertilizers, pesticides and labor. The geographical coordinates of Haining City, China are 30°15′–30°35′ north latitude and 120°18′–120°52′ east longitude (see Figure 1). It belongs to the north subtropical marine humid climate area, with mild climate and abundant rainfall. In general, irrigation water is not needed. Thus, the power consumed for irrigation is not considered herein. This research only considers the agricultural planting link. The diesel consumed in the pre- and post-transportation stages are not calculated within the accounting boundary.

#### 2.2.2. Defining Greenhouse Gases

**Figure 2:**
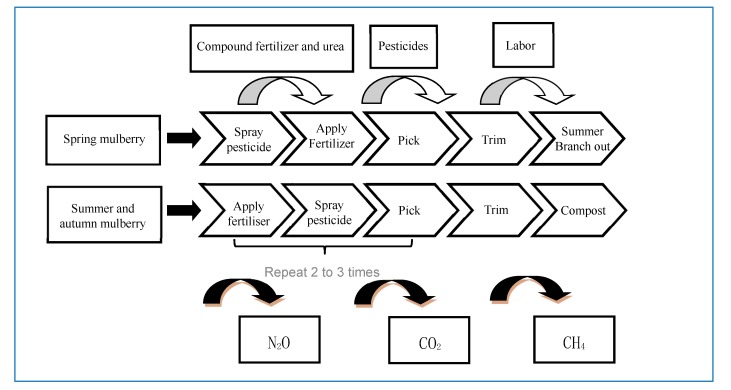
Boundary of carbon footprint accounting system in mulberry planting stage.

Greenhouse gas emissions are divided into direct carbon emissions and indirect carbon emissions. The carbon dioxide produced by workers’ respiration and manure stacking is direct carbon emission. Methane and nitrous oxide are produced by manure during natural stacking. The carbon dioxide produced by pesticide spraying and chemical fertilizer application belong to indirect carbon emission. Therefore, greenhouse gases are defined as carbon dioxide, nitrous oxide, and methane. The boundary of the carbon footprint accounting system in the mulberry production stage is shown in Figure 2.

#### 2.2.3. Calculation Formula

Carbon emissions from agricultural production refer to the total greenhouse gas emissions from the production, transportation, and use of inputs such as electricity for irrigation, chemical fertilizers, pesticides, and agricultural plastic film during the whole production process from sowing to harvesting, as well as the direct N_2_O emissions from farmland soil. According to the production process of mulberry and the research results of Tian Yun [42] and Cheng Kun [43], the calculation formula of carbon emission in the production stage of mulberry was constructed. The calculation formula of carbon emission is as follows:

(1) $$CE=\sum_{i=1}^{n}\;CE_{i}+CE_{N_{2}O}=\sum_{i=1}^{n}\;\sigma_{i}\times C_{i}+C_{N}\times \sigma_{N_{2}O}\times \frac{44}{28}\times 298\times \frac{12}{44}$$

where *CE* is the total carbon emission (kgce/hm^2^), *CE_i_* is the carbon emission of each production input (kgce/hm^2^), *σ_i_* is the carbon emission parameters of each production input *i* (*i* = 1, 2, 3, 4, 5, and 6, respectively denoting artificial, manure, nitrogen, phosphorus, potassium, and pesticide), *C_i_* is the amount of each production input *i*, *CE_N__2__0_* represents carbon footprint caused by direct N_2_O emissions from application of N fertilizer (kgce/hm^2^); *C_N_*, quantity of N fertilizer (kg/hm^2^) applied for mulberry production; *σ_N2O_*, emission factor of N_2_O emission induced by N fertilizer application (tN_2_O–N t^−1^ N fertilizer); 44/28, the molecular weight of N_2_ in relation to N_2_O; 298, net global warming potential (GWP) in a 100-year horizon; 12/44, the molecular weight of CO_2_ in relation to CE [44].

Photosynthesis carbon sink is calculated as follows:

(2) CS=s⋅EP⋅(1−θ)/X

where *CS* is the carbon absorption amount of photosynthesis carbon sink (kgce/hm^2^); *s* is the carbon absorption rate of photosynthesis carbon (kgce/kg), referring to the carbon required for each synthetic unit of organic matter; *EP* is the economic output of crops (kg), which refers to the quality of mulberry leaves with economic use produced during planting and growth; and *θ* is the water content ratio of the economic output part of mulberry, indicating the ratio of crop water content to the whole mulberry leaf quality; *X* is the economic coefficient of mulberry, which is the ratio of the total amount of organic matter produced and accumulated through photosynthesis between the marketable mulberry leaf and the whole growth period of mulberry (including the marketable mulberry leaf, remaining mulberry leaf, branch, shoot, and stem).

Net carbon emission is calculated as follows:

*CE_N_* = *CE* − *CS* (3)

where *CE_N_* is the net carbon emission equivalent (kgce/hm^2^); the net value of the total carbon emission minus the photosynthesis carbon sink is measured, with *CE* as the total carbon emission equivalent (kgce/hm^2^) and *CS* as the photosynthesis carbon sink (kgce/hm^2^).

In the study, the nutrient content of the compound fertilizer (GB15063-2009) used in mulberry production is 45%, that of calcium magnesium phosphate (GB20412-2006) is 12%, that of urea (GB2440-2001) is 46%, and that of ammonium bicarbonate (GB1888-2008) is 17%. According to relevant research results, the parameters of carbon emission and photosynthesis carbon sink are selected herein. According to the actual agricultural production in China, the relevant parameter values in formulas (1)–(3) are shown in Table 1.

### 2.3. Carbon Footprint Assessment Method

With reference to the research results of Song Bo [33] and Tian Yun [42], we evaluate the carbon footprint of mulberry production using four indicators: land carbon intensity, carbon ecological efficiency, carbon production efficiency, and carbon economic efficiency.

#### 2.3.1. Land Carbon Intensity

Land carbon intensity represents the carbon emission per unit planting area of mulberry trees and the carbon emission per square meter soil of the production system. The calculation formula is

*S* = *CE/M* (4)

where *S* is the land carbon intensity (kgce/m^2^), *CE* is the total carbon emission (kgce/hm^2^) and *M* is the land area (m^2^). The large *S* value means a large amount of carbon emission per unit land area.

#### 2.3.2. Carbon Ecological Efficiency

Carbon ecological efficiency refers to the ratio of photosynthetic carbon to total carbon emissions produced in mulberry production. It is one of the indicators used to evaluate the sustainability of mulberry production. The calculation formula is

*KC* = *CS/CE* (5)

where *KC* is the ecological efficiency of carbon and is a dimensionless index; *CS* and *CE* are the carbon sink of photosynthesis (kgce/hm^2^) and total carbon emission (kgce/hm^2^), respectively. *KC* > 1 shows that the carbon emission of mulberry production is less than the carbon sink and that mulberry production have positive externalities to the ecological environment. The larger the value is, the higher the sustainability of the production system will be. Given 0 ≤ *KC* < 1, the greater the carbon emission of mulberry production is, the closer the value of *KC* to 0 will be, and the lower the sustainability of the production system is. When *KC* = 1, the carbon emission of mulberry production is equal to carbon sink; otherwise, the state of the system is neutral.

#### 2.3.3. Carbon Production Efficiency

Carbon production efficiency refers to the ratio of economic output and carbon emission of mulberry. It is an efficiency index used to measure the economic output of unit carbon emission in a mulberry production system. The calculation formula is

*PC = EP/CE* (6)

In the formula, *PC* is the carbon production efficiency (kg/kgce), and *EP* and *CE* represent the economic output (kg/hm^2^) and total carbon emission (kgce/hm^2^) of mulberry, respectively. *PC* reflects the economic output per unit of carbon emission of the production system. A large *PC* value means a high economic output per unit of carbon dioxide emission.

#### 2.3.4. Carbon Economic Efficiency

Carbon economic efficiency is the ratio of the total output value of mulberry to carbon emission. It measures the economic benefits brought by each unit of carbon emission of a mulberry production system. The calculation formula is as follows:

*JC = T/CE* (7)

where *JC* is the carbon economic efficiency (Yuan/kgce); *T* and *CE* are the total output value (Yuan/hm^2^) and total carbon emission (kgce/hm^2^), respectively. *JC* describes the economic benefits per unit of carbon emission of the production system. A large *JC* value indicates great economic benefits per unit of carbon dioxide emission.

## 3. Results and Discussion

### 3.1. Overall Analysis Based on the Carbon Footprint of Mulberry Production in 2014

According to the statistical survey data of the Haining Agricultural Economic Bureau of China, formulas (1)–(3) are used to calculate the carbon footprint and evaluation index values of the mulberry planting area per hectare in 2014 (Table 2).

Table 2 shows that in 2014, the total carbon emissions generated by mulberry trees on 1 hm^2^ land were 5617.92 kgce, including 810.43 kgce (14.43%) for direct carbon emissions and 4807.49 kgce (85.57%) for indirect carbon emissions. Indirect carbon emissions exceed those of direct carbon emissions. The carbon emissions of each production input are in the descending order of nitrogen, manpower, phosphate, manure, potash and pesticide. Among them, the carbon emissions generated by chemical fertilizer are far greater than the sum of the carbon emissions of other production inputs (manpower, manure, pesticides). It is the largest source of greenhouse gas emissions in the section, with a contribution rate of 54.36%, shows that mulberry production is highly dependent on chemical fertilizers because in the mulberry production stage, chemical fertilizers should be applied before and after each picking period. In addition, mulberry has large leaves and high leaf yield. It also requires large amounts of water and chemical fertilizer for cultivation.

In addition, the denitrification of nitrogen fertilizer in the soil will directly release a large amount of N_2_O gas. Compared with other greenhouse gases, N_2_O has the characteristics of high potential for warming, long time in the atmosphere and destruction of the ozone layer, and its negative effects are more obvious [48]. The carbon footprint of soil is 1745.19 kgce, accounting for 31.06% of the total carbon emissions, which is second only to nitrogen fertilizer.

The mechanization degree of mulberry planting industry in Haining is relatively low, and labor input is relatively large. The labor carbon emission is 750 kgce/hm^2^, which is the third largest emission source in mulberry production stage. The carbon emission of manure is 60.43 kgce/hm^2^; manure is mainly used for composting after harvesting in late autumn to increase the organic matter in soil and improve the soil structure. In addition, the heat generated by the fermentation of manure during composting can make the seeds of weeds in the manure of livestock and poultry inactivate, avoid the overgrowth of weeds after application and increase the mulberry output in the next year. The carbon emission of pesticides is 7.81 kgce/hm^2^. Pesticides should be sprayed at all stages to prevent pests because mulberry leaves are good in quality and rich in nutrition and are thus easily damaged by leaf-eating pests.

In spring, the photosynthesis carbon sink is 5066.55 kgce/hm^2^, approximately twice that of in summer and autumn. The total photosynthesis carbon sink in 2014 is 7665.17 kgce/hm^2^, which is more than the total carbon emission. The net carbon emission is −2047.33 kgce/hm^2^. The net carbon emission is negative, and the carbon ecological efficiency is greater than 1, indicating that the carbon emission is less than the photosynthesis carbon sink and that mulberry production has a positive externality to the ecological environment. The carbon intensity of the land is 0.56 kgce/m^2^, signifying that the carbon emission of mulberry production per 1 m^2^ planting area is 0.56 kgce. The carbon ecological efficiency is 1.36, indicating that the carbon sink of photosynthesis is 1.36 unit for every unit of carbon emission produced in mulberry production. The carbon production efficiency is 7.58 kg/kgce, showing that the economic output of 7.58 kg can be obtained for every 1 kgce of mulberry production. The carbon economic efficiency is 9.10 Yuan/kgce, indicating that the economic value of mulberry production is 9.10 Yuan per 1 kgce of carbon emission.

### 3.2. Comparative Analysis of Carbon Footprint of Mulberry Production in Different Years

During mulberry production, farmers in different years have different inputs and outputs. Therefore, the carbon footprint, carbon intensity and carbon efficiency of mulberry production in different years are also different. According to the field survey data obtained from the random sampling of mulberry production in different years, the carbon footprint, photosynthesis carbon sink, carbon intensity and carbon efficiency of mulberry from 2014 to 2016 are calculated using formulas (1)–(3).

As shown in Table 3, the total carbon emissions generated in 2016 were the highest at 6550.73 kgce/hm^2^, followed by 5729.64 kgce/hm^2^ in 2015 and 5617.92 kgce/hm^2^ in 2014. The total carbon emissions in 2016 were significantly higher than those in 2015 and 2014. By comparing the carbon emissions of various production inputs in 2014–2016, it can be seen that the direct carbon emissions in these three years are slightly different. As far as the carbon footprint brought by labor is concerned, because the mulberry planting industry in Haining city adopts the planting mode based on farmers’ families, the annual labor input is almost unchanged, and the annual carbon emission is almost the same, close to 750 kgce/hm^2^. At the same time, with the development of China’s economy and science and technology, the use of manure in Haining City has been kept at a relatively low level, and the fluctuation range of its use is not large.

By comparing the use of production inputs over the past three years, we found that the use of nitrogen fertilizer changed greatly. In 2014, for every 1 hm^2^ mulberry tree put into production, farmers applied 1366.64 kg of nitrogen fertilizer. In 2015, for every 1 hm^2^ mulberry tree put into production, farmers applied 1395.92 kg of nitrogen fertilizer. However, the amount of nitrogen input in 2016 became 1640.73 kg/hm^2^. The increased use of nitrogen fertilizer not only resulted in a significant increase in carbon emissions, but also in N_2_O emissions caused by denitrification in the soil. Total carbon emissions due to increased use of nitrogen fertilizer increased by about 17.54 percent in 2016 compared with 2015. In summary, total carbon emissions in 2016 were significantly higher than in the previous two years, mainly due to increased use of nitrogen fertilizers.

As far as the carbon footprint of chemical fertilizer is concerned, the proportions of chemical fertilizer applications in all years were close, with nitrogen fertilizer as the most dominant, followed by phosphorus and potassium. The main reason is that mulberry leaves grow rapidly in spring, summer and autumn; hence, chemical fertilizers should be applied before and after picking, whereas nitrogen fertilizer can promote leaf hypertrophy. The greatest pesticide carbon footprint was recorded in 2016, followed by those in 2014 and 2015; nevertheless, the overall values were similar. This result is explained as follows. Before the sprouting of mulberry in spring, summer, and autumn, the same dose of pesticide is sprayed to prevent pests. The numbers of trees planted per hectare in all years were also similar, with the average being 12,150 plants/hm^2^.

The carbon footprint evaluation indexes of mulberry production in different years are shown in Table 4. In terms of the annual carbon sink of photosynthesis, the maximum was 7862.21 kgce/hm^2^ in 2015, followed by 7665.17 kgce/hm^2^ in 2014 and 6571.53 kgce/hm^2^ in 2016. The annual carbon sink of photosynthesis in 2016 is significantly lower than the previous two years. The reason is that in the summer and autumn of 2014, mulberry was damaged by mulberry borer pests, the whole process of mulberry production in 2016 was damaged by mulberry borer pests and high temperature, and the mulberry leaf loss was serious. As a result, the photosynthetic carbon sink per hectare in 2014 was lower than that in 2015. Moreover, because spring is the high-yielding period of mulberry production, with the output being much higher than the output value of mulberry in summer and autumn, the photosynthetic carbon sink per hectare in 2016 was significantly lower than that in the previous two years.

Combined with Table 3, we can see that the total carbon emissions generated in 2016 were the most, 6550.73 kgce/hm^2^, followed by 5729.64 kgce/hm^2^ in 2015, and 5617.92 kgce/hm^2^ in 2014. As a result, in terms of net carbon emissions, in 2015, the maximum is −2132.71 kgce/hm^2^, followed by −2047.33 kgce/hm^2^, in 2016, the minimum is −20.80 kgce/hm^2^, and the net carbon emissions are significantly less than the previous two years. The reason is that under the premise of the impact of mulberry leaf yield, the input of fertilizers, manure and pesticides in 2016 was higher than that in the previous two years. Moreover, the two-way surplus of carbon emission and carbon sink of photosynthesis made the net carbon emission in 2016 much lower than that in the previous two years. But in general, the net carbon emissions in 2014–2016 were all negative, indicating that agricultural production can bring positive externalities to the environment.

The land carbon intensity in 2016 was significantly higher than that in 2015 and 2014. In terms of carbon ecological efficiency, it were almost unchanged in 2014 and 2015, and significantly greater than 1, indicating that mulberry production brought significant positive externalities to the environment. In 2016, the carbon ecological efficiency decreased significantly due to the reduction of photosynthesis carbon sink and the significant increase of carbon emission. The value of carbon ecological efficiency is slightly greater than 1, which can still bring positive externalities to the environment. At the same time, due to the impact of natural disasters, the economic output and economic benefits per unit of carbon footprint generated by emissions in 2016 decreased significantly. Overall, the carbon ecological efficiency, carbon production efficiency and carbon economic efficiency in 2015 are the best in three years.

## 4. Conclusions

In general, the total carbon emissions of the mulberry production stage are mainly indirect carbon emissions, and industrial inputs are the main cause of carbon emissions, accounting for 85%–88% of the total carbon emissions. The carbon emissions of each production input were arranged in decreasing order as follows: Fertilizer, labor, manure, and pesticide. The output value of mulberry in spring is larger than that in summer and autumn. In sum, the total carbon emission of mulberry in the production stage is less than the carbon sink of photosynthesis, has positive externalities on the ecological environment, the net carbon emission is negative, the carbon ecological efficiency is greater than 1. The reasonable control of the proportion of ‘industrial inputs’ in mulberry production can reduce the total carbon emissions and improve the positive externalities.

According to the analysis of the carbon footprint of mulberry production in different years, the carbon emission of mulberry in 2016 was the highest at 6550.73 kgce/hm^2^, followed by 5729.64 kgce/hm^2^ in 2015 and 5617.92 kgce/hm^2^ in 2014. The main carbon emission of mulberry in each year originated from chemical fertilizer. In terms of photosynthesis carbon sink, it ranked first in 2015 at 7862.21 kgce/hm^2^. The net carbon emission in 2016 was the lowest, and the carbon production efficiency was inferior. The ecological environment of the mulberry industry can be improved by increasing the carbon sink resources and reducing the pollution of production inputs. An increase in output can also improve the positive externality of carbon emissions, more advanced transplantation and branching techniques can increase the yield of mulberry leaves, this need to be more carefully managed.

The evaluation index analysis shows that the land carbon intensity of mulberry production is small, indicating that the carbon emission per square meter of land is low and that the carbon ecological efficiency value is greater than 1. This result indicates that the total carbon emission of mulberry production is less than the photosynthesis carbon sink, which has a positive externality to the ecological environment.

The scattered planting pattern of farmers is greatly affected by weather, diseases, and insect pests. Despite the guidance of technicians, the farmers still applied fertilizer at will. Advanced technical guidance for farmers’ planting process, reasonable control of the use of ‘industrial inputs’ in the mulberry production process and precise fertilization can reduce the pollution sources of production inputs and effectively reduce the total carbon emissions. Advanced and centralized large-scale planting mode can improve productivity, uniform management allows precise fertilization, reduce carbon emissions and further improve the positive and external carbon emissions of mulberry production.

This paper has potential limitation. The limitation is: the economic coefficient of mulberry used in the photosynthesis carbon sink estimation model of mulberry production process in this paper is taken from the experience value of experts on mulberry production in China. However, China is a vast country, there are some limitations in using the average economic coefficient of Chinese mulberry production to represent the mulberry production level of Haining City. In the future research, we should further build a more targeted economic coefficient value suitable for Haining City.

## Figures and Tables

**Table 1:** Parameters in the formulas of carbon emission and carbon sequestration sunk by photosynthesis in mulberry planting.

Parameter Name	Determination Value	Source Reference
Carbon emission coefficient of manual labor ($\sigma_{1}$)	0.2500 kgce/d	Yang Shihong [45]
Carbon emission coefficient of manure ($\sigma_{2}$)	4.1455 kgce/t	Intergovernmental Panel on Climate Change(IPCC) [46]
Carbon emission coefficient of nitrogenous fertilizer ($\sigma_{3}$)	2.116 kgce/kg	Chen Shun et al. [47]
Carbon emission coefficient of phosphate fertilizer ($\sigma_{4}$)	0.636 kgce/kg	Chen Shun et al. [47]
Carbon emission coefficient of potassic fertilizer ($\sigma_{5}$)	0.180 kgce/kg	Chen Shun et al. [47]
Emission coefficient of pesticide ($\sigma_{6}$)	4.9341 kgce/kg	Tian Yun et al. [42]
Emission coefficient of N_2_O induced by N fertilizer (*σ*_*N*_2_*O*_)	0.01 tN_2_O–N t^−1^ fertilizer-N	IPCC [44]
Photosynthetic carbon absorption rate (s)	0.4500 kgce/kg	Tian Yun et al. [42]
Organism water content ratio (θ)	0.8000	Note ^1^
Economic coefficient (X)	0.5000	Note ^1^

Note ^1^: The water content ratio and economic coefficient of biological organisms are obtained from the average value of that obtained by experts.

**Table 2:** Carbon footprint of mulberry planting stage in 2014.

**Project**	**Direct Carbon Emission**			**Indirect Carbon Emission**						**Total Carbon Emission**
	**Labor**	**Manure**	**Total**	**Fertilizer**			**Pesticides**	**N_2_O**	**Total**	
				**Nitrogenous**	**Phosphate**	**Potash**				
**Carbon footprint per unit area(kgce/hm^2^)**	750.00 *	60.43	810.43	2891.80	151.38	11.31	7.81	1745.19	4807.49	5617.92
**Share of total carbon emissions (%)**	13.35	1.08	14.43	51.47	2.69	0.20	0.14	31.06	85.57	100.00
**Photosynthesis Carbon Sequestration/(kgce/hm^2^)**			**Net carbon emission** **(kg ce·hm^−2^)**		**Land carbon intensity (kg ce·m^−2^)**	**Carbon ecological efficiency**	**Carbon production efficiency (kg·kgce^−1^)**		**Carbon economicefficiency (Yuan·kg ce^−1^)**	
**Spring**	**Summer and autumn**	**Total**	−2047.33		0.56	1.36	7.58		9.10	
5066.55	2598.62	7665.17								

Note: * No consideration is given to gender and age in the calculation of manpower quantities. Assuming that adult labor is homogeneous, a single adult labor force is recorded as 1 labor person per day.

**Table 3:** Carbon footprint of mulberry planting in different years.

Year	Project	Direct Carbon Emission			Indirect Carbon Emission						Total Carbon Emission
		Labor	Manure	Total	Fertilizer			Pesticides	N_2_O	Total	
					Nitrogenous	Phosphate	Potash				
**2014**	**Carbon footprint per unit area** **(kgce·hm^−2^)**	750.00	60.43	810.43	2891.80	151.38	11.31	7.81	1745.19	4807.49	5617.92
	**Share of total carbon emissions (%)**	13.35	1.08	14.43	51.47	2.69	0.20	0.14	31.06	85.57	100.00
**2015**	**Carbon footprint per unit area** **(kgce·hm^−2^)**	750.00	79.00	829.00	2953.75	155.87	4.00	4.43	1782.58	4900.64	5729.64
	**Share of total carbon emissions (%)**	13.09	1.38	14.47	51.55	2.72	0.07	0.08	31.11	85.53	100.00
**2016**	**Carbon footprint per unit area** **(kgce·hm^−2^)**	750.00	70.72	820.72	3471.78	150.33	4.50	8.20	2095.21	5730.01	6550.73
	**Share of total carbon emissions (%)**	11.45	1.08	12.53	53.00	2.29	0.07	0.13	31.98	87.47	100.00

**Table 4:** Evaluation index values of carbon footprint of mulberry planting in different years.

Year	Photosynthesis Carbon Sequestration (kgce·hm^−2^)	Net Carbon Emission (kgce·hm^−2^)	Land Carbon Intensity (kgce·m^−2^)	Carbon Ecological Efficiency	Carbon Production Efficiency (kg·kgce^−1^)	Carbon Economic Efficiency (Yuan·kgce^−1^)
2014	7665.17	−2047.33	0.56	1.36	7.58	9.10
2015	7862.21	−2132.71	0.57	1.37	7.62	9.15
2016	6571.53	−20.80	0.66	1.003	5.57	6.69
